# Prediction of phosphothreonine sites in human proteins by fusing different features

**DOI:** 10.1038/srep34817

**Published:** 2016-10-04

**Authors:** Ya-Wei Zhao, Hong-Yan Lai, Hua Tang, Wei Chen, Hao Lin

**Affiliations:** 1 Key Laboratory for Neuro-Information of Ministry of Education, School of Life Science and Technology, Center for Informational Biology, University of Electronic Science and Technology of China, Chengdu 610054, China; 2 Department of Pathophysiology, Southwest Medical University, Luzhou 646000, China; 3 Department of Physics, School of Sciences, and Center for Genomics and Computational Biology, North China University of Science and Technology, Tangshan 063000, China

## Abstract

Phosphorylation is one of the most important protein post-translation modifications. With the rapid development of high-throughput mass spectrometry, phosphorylation site data is rapidly accumulating, which provides us an opportunity to systematically investigate and predict phosphorylation in proteins. The phosphorylation of threonine is the addition of a phosphoryl group to its polar side chains group. In this work, we statistically analyzed the distribution of the different properties including position conservation, secondary structure, accessibility and some other physicochemical properties of the residues surrounding the phosphothreonine site and non-phosphothreonine site. We found that the distributions of those features are non-symmetrical. Based on the distribution of properties, we developed a new model by using optimal window size strategy and feature selection technique. The cross-validated results show that the area under receiver operating characteristic curve reaches to 0.847, suggesting that our model may play a complementary role to other existing methods for predicting phosphothreonine site in proteins.

Reversible phosphorylation of protein is an important regulatory mechanism that occurs in both prokaryotic and eukaryotic organisms^1^,^2^. It is required in the majority of physiological and pathological processes, such as cell signaling transduction, neural activity, etc. In eukaryotic proteins, more than 30% of proteins are estimated to undergo the reversible phosphorylation^3^. Phosphorylation results in a conformational change in the structure of these proteins in many enzymes and receptors and causes them to become activated or deactivated. Phosphorylation is a process that a phosphoryl group of adenosine triphosphate (ATP) is transferred to some special amino acid residues, thereby generating adenosine diphosphate (ADP)^4^ as shown in Fig. 1. Phosphorylation usually occurs on serine (S), threonine (T) and tyrosine (Y) residues in eukaryotic proteins.

There are several experimental ways to discover protein phosphorylation sites. High throughput Mass Spectrometry (MS) is one of the most popular technique^5^. However, the experimental method is time-consuming and inefficient, thereby a series of excellent algorithms are used in phosphorylation site prediction, such as Artificial Neural Network (ANN)^6^ and Support Vector Machines (SVM)^7^ etc. Some excellent webserver for phosphorylation site predictors have been constructed based on these algorithms. For example, Ingrell *et al*.^8^ developed NetPhosYeast to predict yeast-specific phosphorylation site based on neural network method. Lin *et al*.^9^ developed a rice-specific SVM predictor called Rice_Phospho by combining amino acid occurrence frequency with composition of *k*-spaced amino acid pairs. To identify kinase-specific phosphorylation site, Huang *et al*.^10^ developed a kinase-specific phosphorylation site prediction tool called KinasePhos based on the profile hidden Markov model. Recently, a wonderful web server called GPS (Group-based phosphorylation Predicting and Scoring)^11^,^12^ was constructed to identify ~70 kinds of kinase-specific phosphorylation site with higher prediction robustness. Although there are many available predictors for phosphothreonine site prediction, two essential issues have remained elusive: amino acid sequence window size is a very important factor for pattern prediction, and the selection of optimal window size based on different types of properties may be different; the investigation of position conservation of residues around phosphothreonine may be invaluable for its prediction. However, there are much less concerned with these views in previous work, and the predicted accuracy still has room to grow.

In this paper, we statistically analyzed the distribution of different properties including position conservation, secondary structure, accessibility and some physicochemical properties of the residues around the phosphothreonine and non-phosphothreonine sites. Based on the statistical results, a new model was developed by using optimal window size strategy and feature selection technique. The prediction performance of the proposed model was measured by using auROC value.

## Results and Discussion

In order to achieve an optimal model which can achieve the highest accuracy for predicting phosphothreonine site, it is of great significance to investigate the properties of residues surrounding phosphothreonine. Thus, we statistically analyzed the distribution of different properties including position conservation of residues, secondary structure content, accessibility and some physicochemical properties of the residues surrounding the phosphothreonine site and non-phosphothreonine site.

### The position conservation

**Figure 2:**
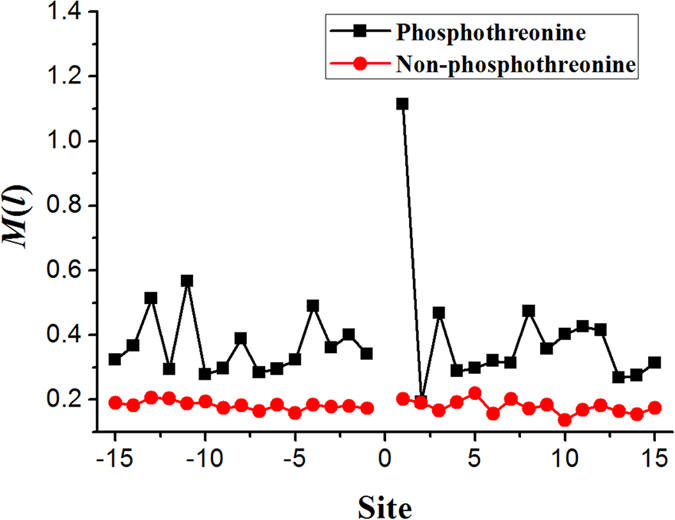
The position conservation *M*(*l*) value around phosphothreonine and non-phosphothreonine sites.

The residues distribution surrounding threonine is an important factor on the phosphorylation of threonine. Thus, the position conservation of residues surrounding the threonine is likely to be a latent and be helpful information to identify phosphorylation sites. To verify our conjecture, the *M*(*l*) value (see Eq (1)) of each position was calculated based on positive and negative datasets, respectively (see Fig. 2). From Fig. 2, we noticed that the *M*(*l*) value of each position in positive dataset is greater than that in negative sample dataset, indicating that these phosphothreonine sites prefer to some special residues.

**Figure 3:**
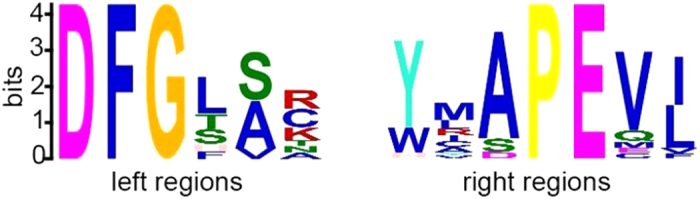
The MEME frequency plot to show consensus motifs of the left and right region around phosphothreonine sites.

Especially, we noticed that the *M(l)* value at +1 site was significantly higher than other sites (Fig. 2), indicating that the first site downstream of threonine plays very important role for phosphorylation. Further analysis shows that the frequency of proline (P) is >25% at the first site downstream of phosphothreonine. We also observed that another five sites (−13, −11, −4, 3, 8) surrounding phosphothreonine site have a higher *M*(*l*) value compared with its neighboring sites. Furthermore, we found that leucine (L), lysine (K) and glycine (G) also have preferences appearing near the phosphothreonine sites, while tryptophan (W), cysteine (C) and histidine (H) present the opposite case around phosphothreonine sites. Thus, we used MEME (Multiple Em for Motif Elicitation)^13^ to generate the consensus motifs around the phosphothreonine sites. As shown in Fig. 3, there are consensus sequences (left: DFG-[SA], E-value: 1.4e-0.09; right: Y-APEV[IL], E-value: 9.6e-045) in the left and right region around phosphothreonine sites, respectively.

### The structural properties

**Figure 4:**
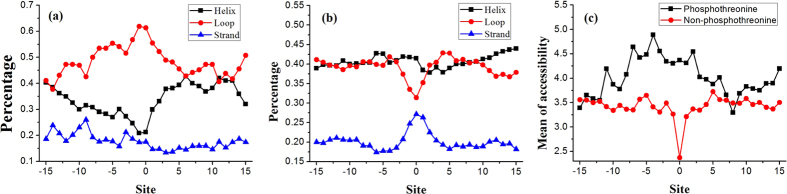
The distribution of the secondary structure and mean of accessibility of each position surrounding the site. (**a**) The distribution of the second structure of the each position around the phosphothreonine site. (**b**) The distribution of the second structure of the each position around the non-phosphothreonine site. (**c**) Mean of accessibility of the each position around positive and negative samples.

Generally, the phosphothreonine sites always located at the surface of proteins. Several studies^14^,^15^,^16^ showed that the phosphorlation sites occur in the loop region. Thus, we performed a serial of analysis to investigate the secondary structure content and accessibility information of the residues around the phosphothreonine site and non-phosphothreonine site. Results are shown in Fig. 4.

From Fig. 4a, we noticed that the residues around the phosphothreonine site do prefer to form loop structure. However, we cannot observe similar phenomena in non-phosphothreonine site (Fig. 4b). Moreover, we found that the closer the site gets to the phosphothreonine site, the greater the frequency of loop structure has and the lower the frequency of helix structure has. Figure 4c shows that the residues around the phosphothreonine site are inclined to have a higher accessibility than the residues around the non-phosphothreonine site. Furthermore, the distributions of secondary structure and accessibility are not strict symmetric flanking from phosphothreonine site (Fig. 4a,c), suggesting that different regions play different roles in phosphorylation of threonine.

### The physicochemical properties

**Figure 5:**
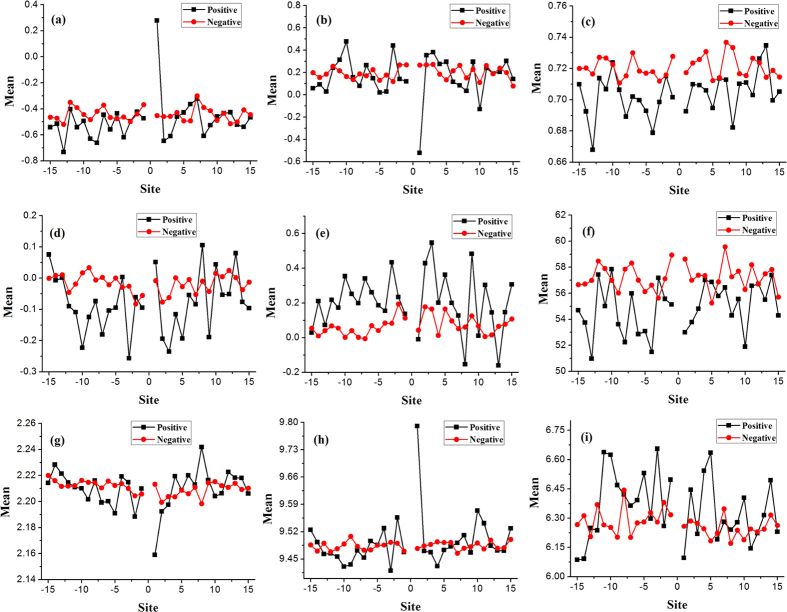
The distribution of average value of residues’ different physicochemical properties in the each position surrounding the site. (**a**) Rigidity, (**b**) Flexibility, (**c**) Irreplaceability, (**d**) Hydrohobicity, (**e**) Hydrophilicity, (**f**) Mass, (**g**) pk1, (**h**) pk2, (**i**) pI.

Physicochemical properties of amino acid play a pivotal role in the protein-related research works for a long time^17^,^18^,^19^. Here, we statistically analyzed the distribution of nine physicochemical properties of residues around the phosphothreonine and non-phosphothreonine site, as shown in Fig. 5. The values of the nine kinds of physicochemical properties have been provided in previous reference^20^. The results showed that, for the nine physicochemical properties, positive samples have larger fluctuation than negative samples. It also shows that the distributions of physicochemical properties flanking the phosphothreonine sites are not symmetrical. Especially, for four properties (rigidity, Flexibility, Pk1 and Pk2), +1 site of phosphothreonine are always dramatically different from other sites, which may be resulted from the phenomenon that +1 site prefer to proline.

### Discrimination phosphothreonine from non-phosphothreonine

Based on the above analysis, we found that the distributions of different properties of residues are not only asymmetric surrounding phosphothreonine, but also dramatically different between phosphothreonine and non-phosphothreonine. These results suggest that the information can be used to identify phosphothreonine.

One may notice that it is unbalance between positive datasets and negative datasets, which will bring biased estimate for a proposed method. Thus, we used clustering algorithm to generate 100 groups of positive sample and 100 groups of negative sample. Then, the first sample in each group are selected as training dataset. Thus, final benchmark dataset includes 100 positive samples and 100 negative samples.

**Figure 6:**
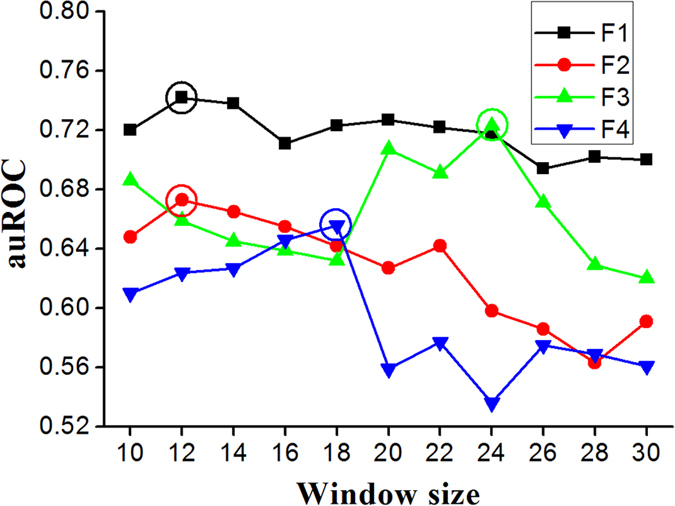
A plot for auROC values with window sizes for four groups of features: position scoring function (F1), secondary structure and accessibility (F2), three kinds of physical properties (rigidity, flexibility and irreplaceability) (F3) and six kinds of chemical properties (hydrophobicity, hydrophilicity, mass, pk1, pk2 and pi) (F4).

A serial of calculation and analysis were performed to obtain the best prediction accuracy. We initially obtain the optimal window size of each set of features as follows. Firstly, four groups of feature sets namely position scoring function (F1), secondary structure and accessibility (F2), three kinds of physical properties (rigidity, flexibility and irreplaceability) (F3) and six kinds of chemical properties (hydrophobicity, hydrophilicity, mass, pk1, pk2 and pi) (F4) were used as features of phosphorylation site prediction, respectively. Secondly, we varied the window size from 10 residues to 30 residues and investigated the accuracy obtained by using SVM in jackknife test. The changes of auROC values with window size for four groups of features were plotted in Fig. 6. Results in Fig. 6 show that the optimal window sizes are 12, 12, 24 and 18 residues, respectively for position scoring function, second structure and accessibility, three kinds of physical properties (rigidity, flexibility and irreplaceability) and six kinds of chemical properties (hydrophobicity, hydrophilicity, mass, pk1, pk2 and pi).

**Figure 7:**
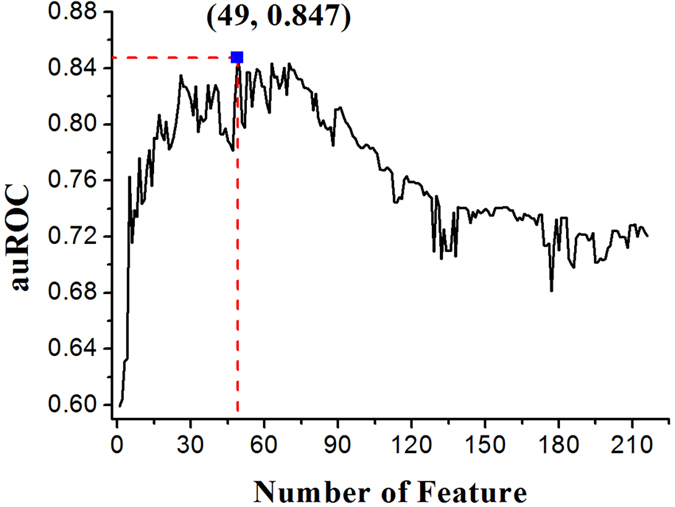
The feature selection results. When the top 49 features were used to perform prediction, the auROC value reached its maximum peak (0.847).

Based on the optimal window sizes for four groups of features, the sample can be formulated as a 216 dimensions vector including 12 dimensions for position scoring function, 12 × 2 = 24 dimensions for secondary structure and accessibility, 24 × 3 = 72 dimensions for three kinds of physical properties (rigidity, flexibility and irreplaceability) and 18 × 6 = 108 dimensions for six kinds of chemical properties (hydrophobicity, hydrophilicity, mass, pk1, pk2 and pi). In order to further improve the accuracy, we used mRMR program^21^ to exclude noise or redundant information. In view of this, we used the incremental feature selection (IFS) to determine the optimal number of feature as described below. The feature subset starts from a feature ranking first in the mRMR feature list. A new feature subset was composed when the second feature of this list was added. We repeated this process until all candidate features are added. In this study, we obtained 216 feature subsets. The prediction performance of each feature subsets was examined by using SVM with jackknife test on the benchmark dataset. We thus plotted a curve in a 2D Cartesian coordinate system with the number of features as its abscissa and the auROC as its ordinate. The maximum auROC corresponds to the peak of the curve which can be easily observed. According to the curve shown in Fig. 7, the auROC reached its peak (auROC = 0.847) when the top ranked 49 features were used.

## Conclusion

In this paper, we statistically analyzed the distribution of the different properties around the phosphothreonine and non-phosphothreonine sites, and found several important features which can contribute the identification of phosphothreonine. In preliminary test, we found that the structural and physicochemical properties do improve the recognition quality of phosphothreonine sites. In the future, we will collect more data and extract more features to construct a predictive model with higher accuracy. Of course, the features can also be used in the prediction of other protein post-translational modification.

## Materials and Methods

### Database

The proteins with experimental-confirmed phosphothreonine in *Homo sapiens* were collected from the Universal Protein Resource (UniProt)^22^. Redundant sequences were removed by using CD-HIT^23^ program with a sequence identity threshold of 30%. The proteins which have three dimension structure information in the Protein Data Bank (PDB)^24^ database were remained. The PDBfinder II database^25^ was used to obtain the corresponding secondary structure and accessibilities of these sequences. Finally, a total of 115 proteins containing phosphothreonine sites were obtained.

Subsequently, we extracted 151 sequences in protein sets. Each sequence is 31-residue long with the experimentally-confirmed phosphothreonines in the center. These sequences are deemed as the positive samples. Correspondingly, the other 31-mer sequences with the center threonine which is unphophorylated are selected as the negative datasets. Thus, we obtained a negative datasets which contains 2158 sequences.

### Feature encoding and selection

#### Position conservation

In order to find potential sequence characteristic of residues around phosphorylation sites (from −15 to +15), we investigated the residue preference at each site by using the conservation formulation defined as follows:


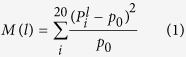


where 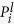 denotes the occurrence frequency of the *i*^th^ amino acid at the *l*^th^ position. *p*_0_ is the background frequency (here *p*_0_ = 0.05). It is obvious that the larger the *M*(*l*) value is, the stronger the conservation of the *l*^*th*^ site is. *M*(*l*) = 0 represents a random distribution of the 20 residues at the *l*^th^ position.

#### The position scoring function

By aligning the training sequences of positive samples, the position weight matrix (PWM) was defined as the following:


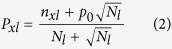


where *n*_*xl*_ denotes the real counts of residue *x* at the *l*^th^ site. *p*_0_ denotes the background frequency of each amino acid in protein sequence (here *p*_0_ = 0.05). *N*_*l*_ denotes the total number of real counts at the *l*^th^ site (the number of sequences). Then, for an arbitrary peptide fragment with 31 residues, the scoring of the *l*^th^ site can be calculated by,


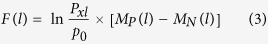


where *M*_*p*_(*l*) and *M*_*N*_(*l*) denote the position conservation at the *l*^th^ site in the positive and negative samples, respectively. The value of *F*(*l*) shows that the degree of sequence close to positive samples.

#### Feature selection

To build an effective prediction model, feature selection is one of the most important steps^26^,^27^,^28^. Generally, not all features have equivalent contribution to the phosphorylation prediction system. Some of features make key contributions, whereas some others are less important. To analyze the features, a mutual information-based feature selection method, Minimal Redundancy Maximal Relevance (mRMR) method^21^, was employed in this study to pick out optimal features.

The mRMR is used to enhance the wrapper feature selection. mRMR can eventually generate two alternative features list, namely MaxRel feature list and mRMR feature list. The MaxRel feature list sorts the features according to their contribution to classification, whereas the mRMR feature list sorts the features by considering not only their contribution to classification but also the correlation between features^29^. In this study, we ranked features according to the mRMR feature list. For the detailed description of mRMR, please refer to Peng *et al*.’s work^21^.

### Model construction

#### Support vector machine

Support vector machine (SVM) is a supervised learning model, which can construct a hyperplane in a high-dimensional space to classify two types of samples and has been widely used in bioinformatics^30^,^31^,^32^. Generally, a good separation is achieved by the hyperplane that has the largest distance to the nearest training-data point of any class (so-called functional margin). Thus, the larger the margin, the lower the generalization error of the classifier is. In this paper, we used LIBSVM 3.20^33^ to perform prediction, which can be freely downloaded from http://www.csie.ntu.edu.tw/~cjlin/libsvm/. The radial basis function (RBF) kernel was selected as kernel function. For achieving the best model, the penalty constant *C* and the kernel width parameter *γ* were tuned in an optimization procedure using a grid search method, of which the search spaces for *C* and *γ* are [2^15^, 2^−5^] and [2^−5^, 2^−15^] with steps of 2^−1^ and 2, respectively.

#### ROC curve

The receiver operating characteristic curve (ROC curve) is a graphical plot to illustrate the performance of a binary classifier system by plotting the true positive rate (*TPR*) against false positive rate (*FPR*) with various threshold settings^34^,^35^. The *TPR*, also called Sensitivity or recall in machine learning, measures the proportion of correctly predicted positive samples. The *FPR*, also known as the fall-out and can be calculated by 1-Specificity, is the proportion of negative samples be predicted as positive samples. TPR and FPR are defined as follows:


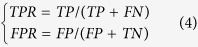


where *TP*, *FP*, *TN* and *FN* denote true positive, false positive, true negative and false negative, respectively. The area under ROC curve (auROC) can better reflect the performance of a classifier. The auROC values ranged from 0.5 to 1, and the larger auROC value is, the better performance is.

## Additional Information

**How to cite this article**: Zhao, Y.-W. *et al*. Prediction of phosphothreonine sites in human proteins by fusing different features. *Sci. Rep.* **6**, 34817; doi: 10.1038/srep34817 (2016).

## Figures and Tables

**Figure 1:**
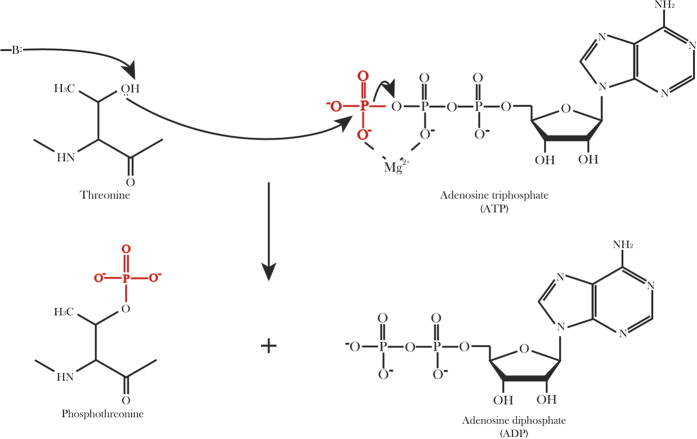
The schematic diagram of threonine phosphorylation. Enzyme-catalyzed proton transfer from the nucleophilic (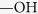) group on threonine attacks the *γ*-phosphate (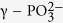) group on ATP, resulting in transfer of the phosphate group to threonine to form phosphothreonine and ADP, while this transfer is facilitated by magnesium (Mg^2+^) and threonine kinase. (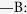) indicates the enzyme base that initiates proton transfer.
